# Construction of the Whole Genome of *Brevundimonas* sp. Strain NIBR11: An Exploration of Bacteria Isolated from Algae in the Nakdong River

**DOI:** 10.1128/mra.00087-23

**Published:** 2023-06-07

**Authors:** Minjae Yu, Jin Young Park, Jaewoong Yu, Jin Nam Kim, Ahhyeon Choi

**Affiliations:** a Department of Bio-Convergence Engineering, Dankook University, Yongin-si, Gyeonggi-do, Republic of Korea; b Biological Resources Research Department, National Institute of Biological Resources, Incheon, Republic of Korea; c eGnome, Inc., Seoul, Republic of Korea; University of Southern California

## Abstract

We report the genome sequence of *Brevundimonas* sp. strain NIBR11. Strain NIBR11 was isolated from algae collected from the Nakdong River. The assembled contig contains 3,123 coding sequences (CDSs), 6 rRNA genes, 48 tRNA genes, 1,623 genes for hypothetical proteins, and 109 genes for proteins with putative functions.

## ANNOUNCEMENT

This study investigated bacteria associated with freshwater algae in the Nakdong River in the Republic of Korea, an essential drinking water source that experiences harmful algal blooms. We aimed to provide foundational data for future algal bloom prevention research, focusing on experimental and analytical aspects of studying bacterial communities in the river. *Brevundimonas* sp. strain NIBR11 was isolated from freshwater algae in the Nakdong River in the Republic of Korea (36°2′26.0″N, 128°15′34.0″E). Algae were collected from the surface (depth of 0 to 15 cm) using a plankton net and were incubated on half-strength Reasoner's 2A (R2A) medium at 20°C to 40°C for 3 days. A single colony isolated by streaking was grown under the same conditions. The 16S rRNA gene of the single colony was amplified with a universal bacterial primer set (27F/1492R), following standard procedures described by Lane (1). This sequence was analyzed by BLASTn searching against the NCBI 16S rRNA gene database and confirmed to be most similar to Brevundimonas staleyi FWC43 (GenBank accession number NR_114710.1), with an identity of 99.3%. The genomic DNA was extracted using the DNA extraction kit (RBC Bioscience, Taiwan) and sheared to >15 kb using g-TUBEs (Covaris, USA). Then, sheared DNA was purified using AMPure XP magnetic beads (Beckman Coulter, Danvers, MA, USA) and measured using a Bioanalyzer (Agilent, USA). The sequencing library was generated using the SMRTBell template preparation kit 1.0 (Pacific Biosciences [PacBio], Menlo Park, CA, USA) and size selected using the Blue Pippin system (Sage Science, Beverly, MA, USA) with a cutoff value of 15 kb. The library was sequenced using the PacBio RS II platform with a SMRT Cell v3.0.

**FIG 1:**
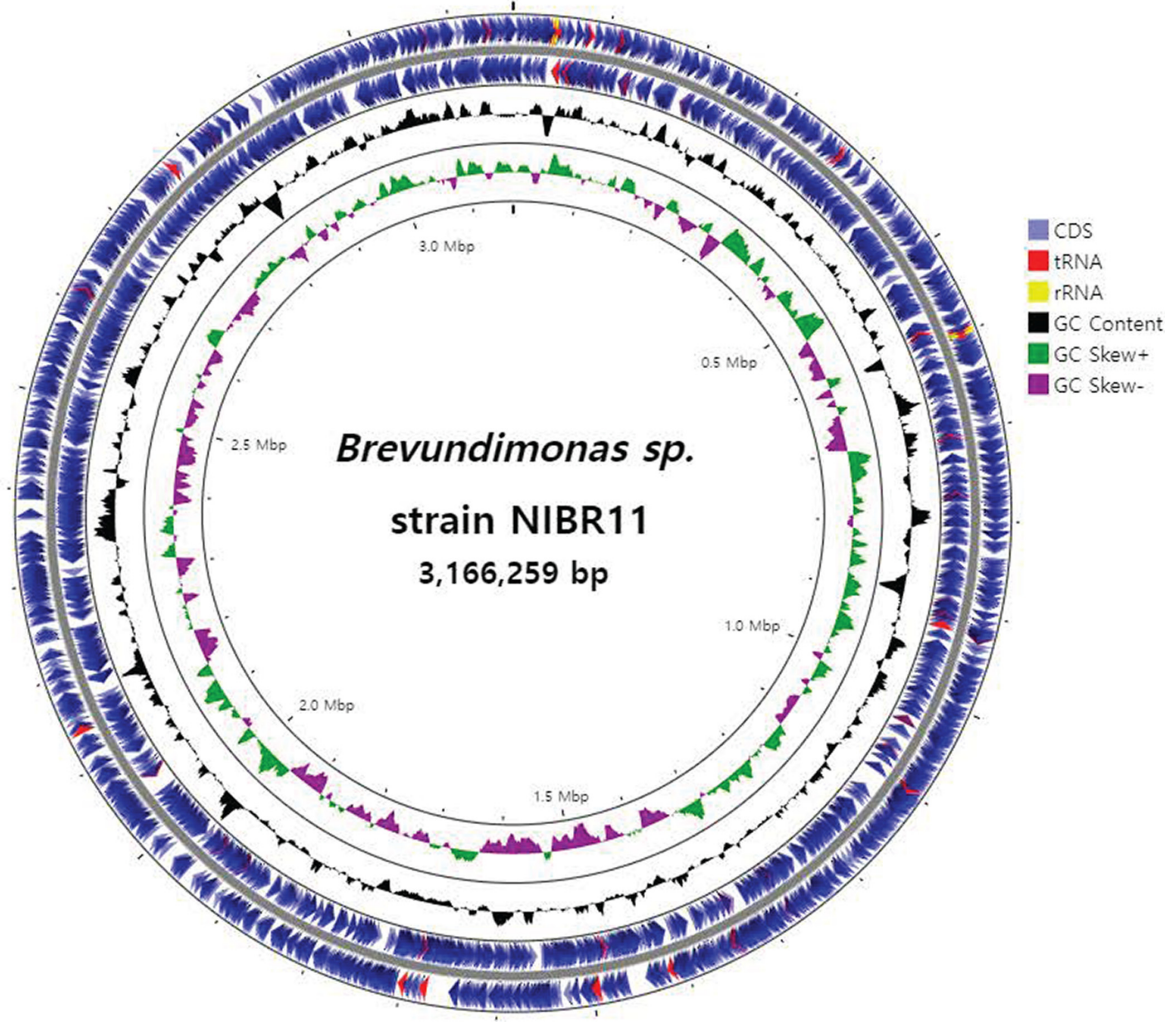
Circular genome map of the *Brevundimonas* NIBR11 chromosome, indicating (outer to inner) CDSs on the forward and reverse strands (blue), tRNAs (red), rRNAs (yellow), GC content (black), positive GC skew (green), and negative GC skew (purple).

The quality of PacBio raw data was evaluated using NanoPlot v1.40.0 (2), and 3,405,221 reads were produced, with an *N*_50_ value of 9,203 bp. Reads of <1,000 bp were filtered using SeqKit v2.3.0 (3). Filtered reads were assembled using Flye v2.9.1-b1780 with the meta option (4). Default parameters were used for all software unless otherwise specified. The contig was rearranged to fix the start position at *dnaA* using Circlator v1.5.5 (5). Finally, one circular contig of 3,166,259 bp was assembled, with a GC content of 67.57%. The genome assembly quality was evaluated using QUAST v5.0.2 (6) and BUSCO v5.2.2 (7). The completeness of the assembled genome was confirmed to be 98.4%. Then, annotation was performed via Prokka v1.14.6 (8), which indicated that the assembly contains 3,123 predicted coding sequences (CDSs), 1,623 genes for hypothetical proteins, 109 genes for proteins with putative functions, 6 rRNA genes, and 48 tRNA genes. The complete genome of strain NIBR11 was visualized with Proksee (https://proksee.ca) (Fig. 1).

For taxonomic classification, the 16S rRNA gene sequence of the assembled genome was used for a BLASTn search against the NCBI 16S rRNA gene database. GTDB-Tk v1.5.0 (9) was used to confirm the taxonomic classification based on 120 bacterial conserved concatenated proteins. In BLASTn analysis, Brevundimonas staleyi FWC43 (GenBank accession number NR_114710.1 [99.29% identity]), Brevundimonas subvibrioides NBRC 16000 (GenBank accession number NR_113834.1 [99.06% identity]), and Brevundimonas subvibrioides ATCC 15264 (GenBank accession number NR_074136.1 [99.10% identity]) were confirmed to exceed the standard identity value of 98.7% with respect to strain NIBR11. From the GTDB-Tk results, it was not possible to identify the taxon at the species level. However, GTDB-Tk assigns NIBR11 to genus *Brevundimonas* and identifies Brevundimonas subvibrioides (GenBank assembly accession number GCA_002280785.1) as the most closely related species, with a FastANI value of 86.29%.

### Data availability.

The complete genome sequence of *Brevundimonas* sp. strain NIBR11 has been deposited in GenBank under the accession number CP115465. The raw data have been deposited in the SRA under the accession number SRR22876607.
